# A rare case of supratentorial ependymosarcoma harboring *ZFTA*::*RELA* fusion

**DOI:** 10.1007/s10014-025-00523-1

**Published:** 2025-11-23

**Authors:** Yuji Matsumoto, Yasuki Suruga, Kaishi Satomi, Yohei Inoue, Yasuhiko Hattori, Joji Ishida, Kazuhiko Kurozumi, Sumihito Nobusawa, Junko Hirato, Takehiro Tanaka, Hiroyuki Yanai, Kana Washio, Koichi Ichimura, Tomotsugu Ichikawa, Yoshihiro Otani, Shota Tanaka

**Affiliations:** 1 Department of Neurological Surgery, Dentistry, and Pharmaceutical Sciences, Okayama University Graduate School of Medicine, Shikata, Kita-Ku, Okayama 700-8558 Japan; 2 Department of Pathology, Faculty of Medicine, Kyorin University, Tokyo, Japan; 3 Department of Neurosurgery, Okayama City Hospital, Okayama, Japan; 4 Department of Neurosurgery, Hamamatsu University Hospital, Hamamatsu, Japan; 5 Department of Human Pathology, Gunma University School of Medicine, Maebashi, Japan; 6 Department of Pathology, Public Tomioka General Hospital, Tomioka, Japan; 7 Department of Pathology, Dentistry and Pharmaceutical Sciences, Okayama University Graduate School of Medicine, Okayama, Japan; 8 Department of Pediatrics, Okayama University Hospital, Okayama, Japan; 9 Department of Neurosurgery, Kagawa Prefectural Central Hospital, Takamatsu, Japan; 10 Department of Pathology, Okayama University Hospital, Okayama, Japan

**Keywords:** Ependymoma, Ependymosarcoma, ZFTA, RELA, Methylation profiling

## Abstract

Ependymosarcoma is an exceedingly rare variant of ependymoma characterized by a mixture of ependymomatous and sarcomatous components. We report a case of supratentorial ependymosarcoma harboring a *ZFTA*::*RELA* fusion in a 10-year-old girl. Histologically, the tumor comprised an ependymomatous component resembling clear cell ependymoma and a sarcomatous component. *ZFTA*::*RELA* fusion was confirmed in both components. Genome-wide methylation profiling classified both components as supratentorial ependymoma, *ZFTA* fusion–positive by the German Cancer Research Center (DKFZ) CNS tumor classifier v12b8. However, their copy number alteration profiles were distinct. The ependymomatous component exhibited a gain of chromosome 1q and a loss of chromosomes 1p, 9, and 19q, while the sarcomatous component showed a loss of chromosome 14. These findings suggest that both components may have differentiated from a common precursor despite their distinct morphologies. The patient underwent gross total resection followed by adjuvant chemoradiotherapy and remains recurrence-free eight years post-treatment. Further investigation of additional cases is warranted to better understand the pathogenesis of this rare tumor.

## Introduction

Ependymomas arising in the supratentorial region affect both pediatric and adult populations, comprising approximately one-third of all intracranial ependymomas [1]. The latest World Health Organization (WHO) Classification of Tumors of the Central Nervous System (CNS5) recognizes two molecularly defined subtypes of supratentorial ependymoma: one harboring a *ZFTA* gene fusion (formerly known as *RELA* fusion), which accounts for the majority of cases, and another characterized by the presence of a *YAP1* gene fusion [1]. The standard treatment is surgical resection with maximal safety, followed by adjuvant radiation therapy. Effective chemotherapy regimens for these tumors have not yet been established [2, 3].

The term “ependymosarcoma” was proposed by Rodriguez et al. in 2008 upon describing 11 cases of ependymal tumors exhibiting sarcomatous changes [4]. Since then, 24 cases have been reported in the literature [4–16]. Recent advances in genome-wide DNA methylation profiling have emerged as a powerful tool, complementing the classification of central nervous system tumors [17]. While several studies have described the genetic characteristics of ependymosarcoma, insights from genome-wide methylation profiling have not yet been reported. Here, we present a case of supratentorial ependymosarcoma harboring a *ZFTA*::*RELA* fusion. Methylation profiling revealed that both the ependymomatous and sarcomatous components were classified as supratentorial ependymoma, ZFTA fusion–positive by the German Cancer Research Center (DKFZ) CNS tumor classifier v12b8; however, they exhibited distinct copy number alteration profiles.

### Clinical summary

**Fig. 1:**
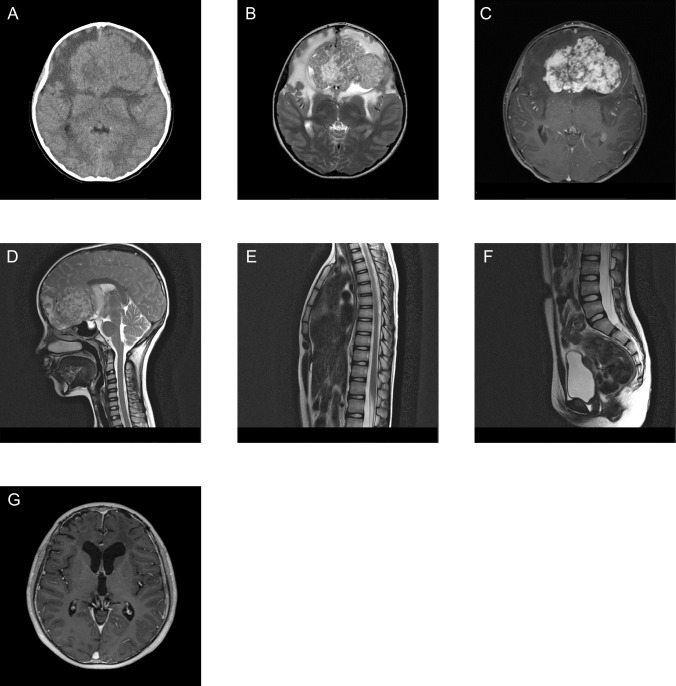
Head computed tomography (CT) scan showing a bilateral frontal mass without calcification **A**. Head magnetic resonance (MR) imaging demonstrates significant peritumoral edema on T2-weighted images and heterogeneous enhancement on gadolinium-enhanced T1-weighted images **B**, **C**. Whole-spine MR imaging shows no evidence of spinal dissemination **D**-**F**. A gross total resection was confirmed on gadolinium-enhanced T1-weighted images (G)

A previously healthy 10-year-old girl presented with a 1-month history of headache and vomiting. The patient had also shown personality changes over the past year. Neurological examination revealed disorientation, cognitive impairment, and olfactory dysfunction. Head computed tomography (CT) revealed a bilateral frontal mass with peritumoral edema (Fig. 1A), without evidence of calcification. Head magnetic resonance (MR) imaging showed marked peritumoral edema on T2-weighted images and heterogeneously enhancement on gadolinium-enhanced T1-weighted images (Fig. 1B, C). No spinal dissemination was observed on whole-spine MR imaging (Fig. 1D–F). A gross total resection was performed (Fig. 1G), and the postoperative course was uneventful. The patient received adjuvant proton beam therapy with a total dose of 59.4 Gy (1.8 Gy/fraction), following four cycles of chemotherapy with vincristine, doxorubicin, cyclophosphamide, ifosfamide, and etoposide (VDC-IE). The patient remains well without the additional adjuvant treatment and has shown no evidence of tumor recurrence on brain MR imaging for eight years.

### Pathological and molecular findings

**Fig. 2:**
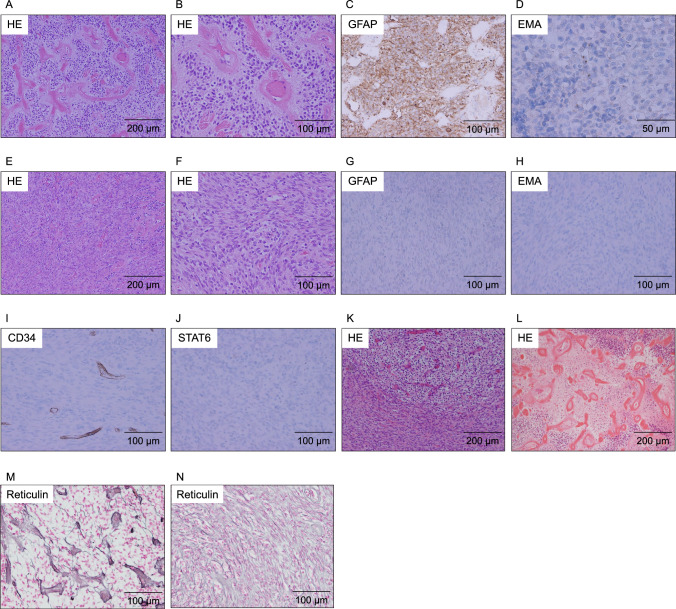
Histologically, the ependymomatous component showed a dense proliferation of clear cells and sclerotic capillaries, forming structures resembling perivascular pseudorosettes **A**, **B**. Immunohistochemical staining of the ependymomatous component revealed positivity for glial fibrillary acidic protein (GFAP) **C** and dot-like staining patterns with epithelial membrane antigen (EMA) **D**. The sarcomatous component exhibited a dense proliferation of spindle-shaped tumor cells with abundant capillaries **E**, **F**. The sarcomatous component was negative for GFAP, EMA, CD34, and STAT6 on immunohistochemical staining **G**-**J**. The two components were spatially distinct with a clearly demarcated border between the ependymomatous and sarcomatous regions **K**. Focal necrosis was present in the ependymomatous component **L**. Reticulin staining demonstrated fibers in both the ependymomatous **M** and sarcomatous **N** components, with the sarcomatous component showing particularly abundant reticulin fibers

**Fig. 3:**
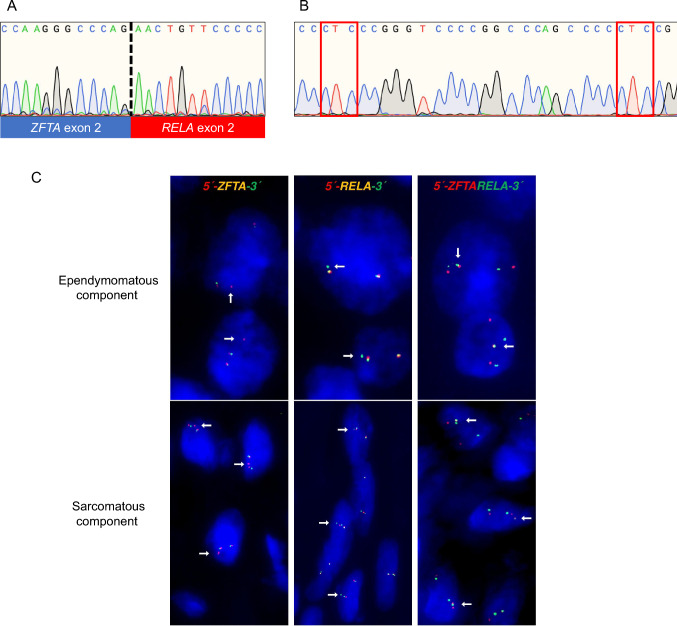
Sanger sequencing using DNA extracted from bulk fresh frozen specimens demonstrated *ZFTA*::*RELA* fusion **A**, while no TERT promoter mutations were identified **B**. **C** The upper and lower panels are for the ependymomatous and sarcomatous components, respectively. Fluorescence in situ hybridization (FISH) analysis using break-apart *ZFTA* or *RELA* probes revealed rearrangement of each gene with isolated red or green signals, respectively (arrow). FISH analysis with 5´-*ZFTA* and *RELA*-3´ probes reveals a fused signal pattern in both components (arrow)

Microscopic histopathological examination revealed that the tumor was composed of two distinct components. One component consisted of densely intermingled clear cells with intervening sclerotic capillaries and exhibited features resembling perivascular pseudorosettes (Fig. 2A, B). In this component, immunohistochemistry demonstrated glial fibrillary acidic protein (GFAP) staining (Fig. 2C) and dot-like inclusion with epithelial membrane antigen (EMA) (Fig. 2D). This component resembled clear cell ependymoma, and given the supratentorial location in a pediatric patient, a *ZFTA*-fusion ependymoma was suggested. The second component showed a dense proliferation of spindle-shaped tumor cells with abundant capillaries and was negative for GFAP, EMA, CD34, and STAT6 on immunohistochemistry (Fig. 2E–J), consistent with a sarcomatous phenotype. These two components were spatially distinct within the tumor, and the border between them was clearly demarcated (Fig. 2K). Mitotic figures were rarely observed in both components, but necrosis was present in the ependymomatous component (Fig. 2L). Reticulin fibers were observed in both the ependymomatous (Fig. 2M) and sarcomatous components (Fig. 2N); however, the sarcomatous component exhibited particularly abundant reticulin fibers. *ZFTA*::*RELA* fusion was verified by reverse transcription polymerase chain reaction and Sanger sequencing from bulk fresh frozen specimens (Fig. 3A), but no *telomerase reverse transcriptase (TERT)* promoter mutations were identified (Fig. 3B). Fluorescence in situ hybridization (FISH) validated *ZFTA*::*RELA* in both components, leading to the diagnosis of ependymosarcoma (Fig. 3C).

**Fig. 4:**
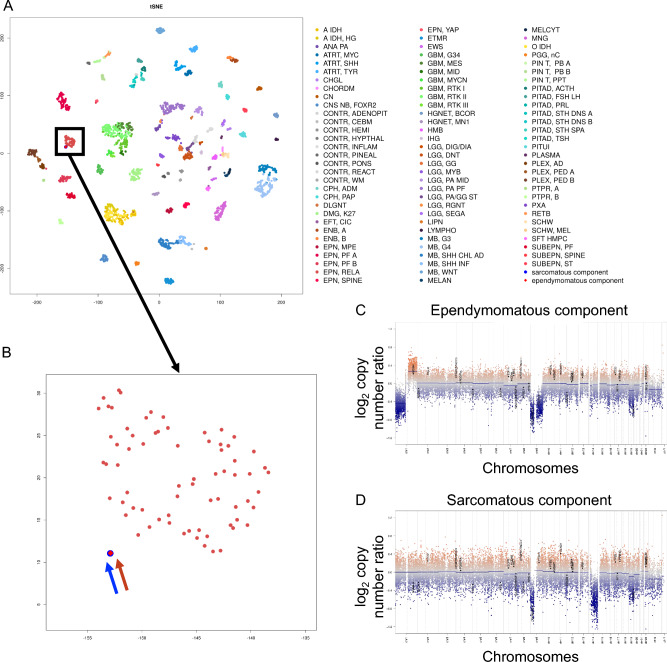
A t-distributed stochastic neighbor embedding (t-SNE) analysis was performed using 2,801 reference cases from the study by Capper et al. **A**, **B**. The red arrow indicates the ependymomatous component, and the blue arrow indicates the sarcomatous component **B**. Distinct copy number alterations were observed, the ependymomatous component exhibiting a gain of chromosome 1q, and a loss of chromosomes 1p, 9, and 19q **C**, while the sarcomatous component showed a loss of chromosome 14 **D**

To further characterize the tumor, DNA was extracted from formalin-fixed, paraffin-embedded (FFPE) samples of the ependymomatous and sarcomatous components for DNA methylation profiling. Both components were classified as supratentorial ependymoma, ZFTA fusion–positive by methylation class family (calibrated scores: ependymomatous component: 0.90, sarcomatous component: 0.99) using the DKFZ CNS tumor classifier v12b8. At the methylation subclass level, the ependymomatous component was classified as ZFTA-RELA fused, subclass A (calibrated score: 0.70), and the sarcomatous component as subclass D (calibrated score: 0.98). A t-distributed stochastic neighbor embedding (t-SNE) analysis using 2,801 Department of Pathology, Okayama University Hospital, Okayama, Japannce cases from the study by Capper et al. [17] revealed that both components clustered closely within the Ependymoma, *RELA* fusion–positive group (Fig. 4A, B). However, their copy number profiles exhibited distinct patterns: the ependymomatous component harbored a gain of chromosome 1q and a loss of chromosomes 1p, 9, and 19q (Fig. 4C), while the sarcomatous component showed a loss of chromosome 14 (Fig. 4D). Additionaly, both the ependymomatous and sarcomatous components showed *CDKN2A/B* homozygous deletions (Fig. 4C, D).

### Discussion

Ependymosarcoma, an extremely rare variant of ependymoma, has been reported in fewer than 25 cases to date [4–16]. These tumors present across a broad age range (2–63 years), with a median age of 32 years. There is a slight predominance of female patients, with 13 females compared to 11 males. The majority of tumors were located in the supratentorial and extraventricular regions. While 13 out of 24 cases arose de novo, one developed upon recurrence after gross total resection, and 10 emerged as recurrent tumor components following radiotherapy. Ependymosarcomas are highly aggressive tumors typically treated with gross total surgical resection followed by radiotherapy and chemotherapy. Although the limited number of ependymosarcoma cases hinders prognostic analysis, more than half of patients with available follow-up data have died from the disease. According to the literature, one patient has survived without recurrence for 12 years after initial gross total resection and subsequent radiotherapy. The epidemiology of this case is consistent with previously reported ependymosarcomas. Although there is no established standard treatment for ependymosarcomas, the favorable outcome in this case may be attributed to the gross total resection followed by intensive adjuvant therapy.

Although some studies have shed light on the molecular features of ependymosarcoma, these tumors have not yet been fully characterized. Rodriguez et al. described various chromosomal imbalances, including gains of 1q, deletions of 22q and 6p, monosomy 18, and polysomies/polyploidy, observed in both the mesenchymal and glial components of ependymosarcomas [4]. Tabarrah et al. reported a case of ependymosarcoma with a t(1;19)(q12;p13) and increased ploidy within the sarcomatous component. They hypothesized that this ploidy amplification might partially account for the morphological diversity seen at the genetic level [14]. Brügger et al. reported that ependymomas with mutations in the TERT promoter are more susceptible to developing into ependymosarcoma. However, they postulated that the TERT mutation is likely a later event in tumor development and not the primary genetic driver underlying the initial tumorigenesis [18]. Previous reports attempting to analyze fusion genes in three cases confirmed the presence of *ZFTA* fusion in both the sarcomatous and ependymomatous components [6, 11].

In the present case, genome-wide methylation profiling of both components revealed novel molecular features of ependymosarcoma. Recent advances in genome-wide DNA methylation profiling have emerged as a powerful tool that offers a complementary approach to classifying central nervous system tumors [17, 19, 20]. The potential of DNA methylation profiling to provide diagnostic and prognostic information beyond histology has been demonstrated for ependymoma as well [21–23]. In the present case, both the ependymomatous and sarcomatous components were classified as supratentorial ependymoma, ZFTA fusion–positive at the methylation class family level. However, the ependymomatous component was further classified as ZFTA-RELA fused, subclass A, while the sarcomatous component was classified as subclass D. Interestingly, these results align with a recent report by Zheng et al., who described that supratentorial ependymoma, ZFTA fusion–positive, subclass D (corresponding to their cluster 3) harboring canonical *ZFTA*::*RELA* fusion is characterized by very unusual histology, often with mesenchymal or sarcomatous aspects [23]. It is noteworthy that the copy number profiles of the two components displayed distinct patterns: the ependymomatous component exhibited a gain of chromosome 1q and a loss of chromosomes 1p, 9, and 19q, whereas the sarcomatous component was characterized by a loss of chromosome 14. Together with the t-SNE analysis showing that both components were positioned at nearly identical coordinates, these results suggest that despite differing morphologies, both components may have differentiated from a common precursor cell. The gain of chromosome 1q and losses of chromosomes 1p, 9, and 19q may have driven the development of the ependymomatous component, while the loss of chromosome 14 may have contributed to the differentiation of the sarcomatous component.

The diagnosis of ependymosarcoma remains highly challenging due to its rarity; however, the recently provisionally proposed “ependymoma-like tumors with mesenchymal differentiation (ELTMD)” should be considered in the differential diagnosis [24]. ELTMD has been reported in both pediatric and adult populations. Histologically, the major components of ELTMD resemble embryonic tissues. These components form well-defined tumor cell nests consisting of small, uniform cells with a high rate of proliferation, as well as spindle-shaped mesenchymal components with a sarcoma-like appearance ranging from low to high grade. The embryonic-appearing components exhibit minimal ependymal differentiation, characterized by the presence of tubular structures and positive staining for CAM 5.2 and EMA. From a genetic standpoint, ELTMDs are characterized by harboring *C11orf95*-*NCOA1/2* or -*RELA* fusion and stable chromosome profiles. Both ependymosarcoma and ELTMD are characterized by a mixture of ependymomatous and sarcomatous components harboring *ZFTA* fusion; however, they exhibit distinct differences in the following characteristics: (1) ELTMDs exhibit well-delineated nests composed of embryonal-appearing cells that are diffusely and strongly positive for CAM5.2, a feature not observed in ependymosarcomas; (2) ELTMDs lack perivascular pseudorosettes, a histological hallmark of ependymomas; (3) The embryonal-appearing components in ELTMDs lack clear cell morphology with branching vessels, which are often observed in supratentorial ependymomas with *ZFTA*::*RELA* fusion; (4) ELTMDs display stable chromosomal profiles, whereas ependymosarcomas exhibit abundant copy number changes.

In conclusion, we present a case of ependymosarcoma, an extremely rare tumor characterized by a mixture of ependymomatous and sarcomatous components, harboring the *ZFTA*::*RELA* fusion. Although the molecular features of ependymosarcoma have not been fully elucidated, genome-wide methylation profiling in this case revealed that both components were classified as supratentorial ependymoma, ZFTA fusion–positive, yet exhibited distinct copy number profiles. These findings suggest that both components may have differentiated from a common precursor cell. Further clinicopathological and molecular analyses of additional cases are crucial for deepening our understanding of these tumors and improving patient outcomes for this challenging diagnosis.

## Data Availability

The datasets generated and/or analyzed during the current study are available from the corresponding author on reason able request.
